# Effects of a Novel Gel Formulation of Dog Appeasing Pheromone (DAP) on Behavioral and Physiological Stress Responses in Dogs Undergoing Clinical Examination

**DOI:** 10.3390/ani12182472

**Published:** 2022-09-19

**Authors:** Ivana Puglisi, Marisa Masucci, Alessandro Cozzi, Eva Teruel, Michele Navarra, Santa Cirmi, Maria Grazia Pennisi, Carlo Siracusa

**Affiliations:** 1 Department of Veterinary Sciences, University of Messina, 98168 Messina, Italy; 2 Research Institute in Semiochemistry and Applied Ethology (IRSEA), 84400 Apt, France; 3 Department of Chemical, Biological, Pharmaceutical and Environmental Sciences, University of Messina, 98168 Messina, Italy; 4 Department of Clinical Sciences and Advanced Medicine, School of Veterinary Medicine, University of Pennsylvania, Philadelphia, PA 19104, USA

**Keywords:** dogs, dog appeasing pheromone, stress, veterinary visit

## Abstract

**Simple Summary:**

Safeguarding the health of dogs presupposes undergoing regular veterinary visits (VVs). However, the VV can be emotionally challenging, thus impairing the welfare of the patient, also reducing dog and owner compliance. The fear and anxiety experienced by the dogs evokes behavioral and physiological stress responses for adaptive purposes. It is therefore desirable to lower the stress from the visit. With this intent, we tested a gel formulation of a synthetic analogue of the appeasing pheromone secreted by bitches, the dog appeasing pheromone (DAP), as a situational support to improve the perception of the stay in the waiting room and the physical examination. In the waiting room, the dogs exposed to DAP exhibited changes in their behavior, namely significant decrease in lip licking, increase in panting, and nearly significant reduction of low body postures. On the examination table, neither behavioral nor physiological differences were found. DAP did not alter markedly the stress behavior and physiology of dogs during a VV, maybe due to a stress level exceeding the potential efficacy of the product. However, the change of a few stress-associated behaviors suggests that DAP could contribute to improving the welfare of dogs staying in the waiting room before the physical examination.

**Abstract:**

The veterinary visit is necessary for safeguarding the health of dogs, but it can be stressful and threaten both the welfare of the patient and the accuracy of the examination. This randomized, triple-blind, placebo-controlled, crossover study aims at evaluating how dog appeasing pheromone (DAP) in a novel gel formulation influences the behavioral and physiological stress responses of 28 dogs undergoing a standardized clinical examination, while staying in the waiting room (WR) and visited in the examination room (ER). Behavioral responses were studied through behavioral categories and subjective scales (WR and ER). Autonomic response considered heart rate (WR and ER), blood pressure (WR and ER), respiratory rate (ER), and rectal temperature (ER). Neuroendocrine response considered salivary cortisol (WR and ER). In the waiting room, the use of DAP was associated with a significant reduction of lip licking (*p* = 0.0189), an increase in panting (*p* = 0.0276), and a reduction close to significance (*p* = 0.0584) of low body postures. No significant differences were observed within the physiological responses. In the examination room, neither behavioral nor physiological differences were found.

## 1. Introduction

The concept of welfare embraces various aspects of the life of an animal, including mental and physical health, the capability of adapting to an artificial environment, and the presence of positive feelings [1]. It is more common to measure welfare by analyzing poor welfare rather than good welfare indicators, being more evident and easily measurable [2], so stress evaluation can provide useful information about the welfare of dogs in the veterinary practice.

Stress is defined as the biological response elicited when an individual faces a stimulus, called stressor, perceived as threatening its homeostasis. This response consists of a combination of four quantifiable defense processes: behavioral, autonomic, neuroendocrine, and immune [2,3]. The impact of a stressor on an individual depends on the stimulus itself, the genetics of the subject, and what he or she learned from previous exposures [3,4,5]. This variability is a reflection of the complexity of the underlying biological stress response [2,3,6] and underscores the need for a multimodal approach to the study of stress through the use of multiple assessment criteria and biomarkers [2,6,7,8].

The behavioral stress response in dogs is manifested as aggression, escape, tonic immobility, or displacement gestures. The response is determined by an analysis of the stimulus, environmental options, physical abilities, and past experiences [9]. Among the physiological stress responses, the activity of the autonomic nervous system (ANS) and the hypothalamic-pituitary-adrenal cortex (HPA) axis allows differentiating the response to an acute stressor from a medium- to long-term response [2,10]. The up-regulation of the sympathetic branch of the ANS occurs within 1 or 2 s from the perception of the stimulus and causes the increase of circulating catecholamines for about 2 min [2], leading to a transient increase in heart rate, blood pressure, respiratory rate, and body temperature in dogs [2,11,12,13,14]. After a few minutes from the perception of the stressor, the activation of the HPA axis causes the release of cortisol into the bloodstream [2,15]. Cortisol circulates for up to one hour from the end of the exposure to the stimulus, producing lasting effects on the body [2,3,4,15,16]. There is a significant correlation between the levels of cortisol in blood and saliva in the dog [16,17,18,19], so both substrates provide useful information for short-term stressful situations [2]. Saliva collection is, however, less invasive than blood collection and saliva cortisol measurement is often preferred [8,11,20,21].

The veterinary visit is an indispensable medical procedure but can be a very unpleasant experience for the patient. Just approaching and staying in the waiting room of a veterinary clinic can cause discomfort to dogs and trigger stress-related behaviors as well as increases in heart rate and serum cortisol levels [22,23,24]. However, the physical examination was found to be particularly stressful for dogs [25,26,27,28,29,30,31,32], eliciting fear-related behaviors; an increase in heart and respiratory rates and body temperature; and increased levels of salivary and urinary cortisol. Questionnaires filled by owners or veterinarians have also been used to assess the stress experienced by dogs during various stages of the veterinary visit, confirming that the latter is indeed a source of stress for the patient [24,33,34]. Behavioral and physiological stress-related responses can mask or worsen some clinical signs and interfere with the diagnostic workup [35,36].

Minimal-stress handling (MSH) of the veterinary patient and a dog-friendly environment have been proposed as effective strategies to minimize the stress experienced by the veterinary patient [8,37,38]. The use of the Dog Appeasing Pheromone (DAP), a synthetic analog of the appeasing pheromone secreted by the sebaceous glands of the intermammary sulcus of nursing bitches that has a calming effect on both puppies and adults [39,40], has also been proposed to decrease stress during veterinary procedures as an alternative or complement to pre-visit medication [38,41,42]. The main benefits of this therapeutic approach consist of its safety, the absence of adverse effects and undesired drug interactions, and its ease of administration. It has been shown that different formulations of DAP produce a calming effect in dogs facing several stressful situations [43,44,45,46,47,48], and something is known about the potential and limitations of its use during the veterinary visit. It has been found that DAP helped reduce the anxiety of veterinary patients both in the waiting and the examination room [49]; improved the recovery and welfare of dogs undergoing surgery [50]; and reduced various separation-related behaviors in hospitalized dogs [51]. More information, however, is needed on the efficacy of DAP in reducing stress-related behaviors during the veterinary visit, including the handling and restraint of a patient.

This study aims to evaluate the efficacy of a gel formulation of DAP, applied to the hands of all people who came into contact with the dogs, as a situational support for reducing the activation of the behavioral, autonomic, and neuroendocrine stress response in dogs undergoing a standardized veterinary examination. We hypothesize that DAP will lower the stress experienced by the dogs during their stay in the waiting room and during handling on the examination table.

## 2. Materials and Methods

### 2.1. Animals

Twenty-eight dogs were enrolled based on the following criteria: age between 1 and 9 years, intact females neither pregnant nor in estrus, no history of severe aggressive behaviors (attempting to bite, biting the handler) during previous physical examinations, good general health (including the absence of oral mucosa lesions), no current pharmacological treatments. Any previous medication must have been suspended or discontinued at least one week before the collection of data for this study. In the case of treatment with corticosteroids, the washout period was extended to one month to exclude potential interferences with the patient’s plasma cortisol levels [52,53] and influence on dog behavior [54].

### 2.2. Experimental Design

The study was performed during a time period of four months at the Department of Veterinary Sciences of the University of Messina (Italy) and consisted of a randomized, fully blinded, placebo-controlled cross-over design trial. The dogs were examined twice, with an interval of 2–4 weeks between visits. The animals were exposed to DAP or a placebo during each of the two visits, according to a randomization list. A compounded formulation of DAP and placebo were provided by IRSEA Research Institute (Quartier Salignan, 84400 Apt, France). The experimenters were blinded and not aware of the specific treatment applied. The two treatments were dispensed as a gel formulation in opaque dispenser bottles. Each bottle was labeled with the letter “A” or “B”. The appearance, texture, and absorption time (30–60 s) of the gel contained in the bottles were consistent between the two treatments. The owner and the veterinarian handling the dog during the physical examination applied the gel to their hands, in the waiting and examination room, respectively, as described below. The composition of the two treatments is summarized in Table 1.

Three experimenters, all veterinarians, were involved in the data collection. One experimenter, Investigator A, was consistently in charge of videotaping the dog in the waiting room and the dog-handler interaction during the veterinary visit. Investigators B and C, who had similar physical traits, rotated to administer the physical examinations on different days of the study. However, each dog was consistently visited by the same investigator. The physical examinations were performed at the same time (±2 h) for every dog, with the animals fasting [55] for at least 2 h to prevent possible contamination of the saliva used for measuring cortisol. We chose relatively short fasting preceding the visit to avoid influencing the cognitive [56] and emotional states of the dogs [57,58]. It was recommended to avoid drinking during the transportation to the clinic and immediately before the visit. The owners were also advised to not involve their dogs in any intense physical activity the 48 h before the visit, because this may have influenced the concentration of cortisol [59,60,61,62].

### 2.3. Behavioral Data Collection

Behavioral data were recorded with a digital videocamera (Handycam^®^ DCR-SX33E, Sony, Minato, Tokyo, Japan). A reference ethogram, consisting of 6 behavioral categories and 16 patterns, was compiled for the behavioral analysis (Table 2) [7,27,50,63,64,65].

In order to determine whether a behavior was displayed conspicuously in the context of the study, we performed preliminary observations on a convenience sample of 8 dogs (different from the dogs included in the final study presented here). Behaviors observed in more than 25% of the 8 dogs were then selected and measured using their frequency or duration in the final study (Table 3). Each behavior that was observed conspicuously (see below) was measured using its frequency or duration. In order to avoid missing data from behaviors that were not conspicuously observed, we then grouped all behaviors in separate categories by their function (see Table 2). These behavioral categories were then measured using a one-zero scoring method.

The behavior of each dog was recorded for 3 min in the waiting room and in the examination room respectively. For the analysis of both the conspicuous behaviors and the functional categories, each 3-min recording was divided into sampling intervals with the following pattern: 10” observation–20” pause–10” observation and so on, for a total amount of six-time intervals of observation. Conspicuous behaviors were measured continuously during the selected sampling times and their frequency or duration was recorded. For the functional categories, the behaviors included in each category were instead registered with a one-zero sampling method. The total occurrences of each behavior (a value between 0 and 6) were added together and this value was then divided by the total number of behaviors in a category. The final resulting value was considered the mean occurrence of that specific functional category.

In addition, four-point Likert-type scales were used by the investigator involved in the clinical examination to evaluate the veterinarian’s perception of the ease of examination and the stress experienced by the patient. In particular, the veterinarian attempted to quantify stress-related behaviors manifested in a freeze-type response that is difficult to quantify, e.g., stiffness or subtle trembling [7,27,31,66] (Table 4).

### 2.4. Autonomic Data Collection

The blood pressure of the dogs was measured following the ACVIM guidelines [67] and using a high-definition oscillometry unit (Vet HDO^®^, DVM Solutions, San Antonio, TX, USA). Minimal-stress handling was used to minimize the impact on cardiovascular performance. The investigator sat on the floor with the dog and its owner, who was allowed to pet the dog if desired [29,68,69,70,71]. Heart and respiratory rates were also collected during the physical examination. The rectal temperature was taken using a lubricated electronic rectal thermometer (SOLUTION-Italy TD004^®^, Vega Technologies Inc., Dongguan, China).

### 2.5. Neuroendocrine Data Collection

A saliva sample was collected using a Salivette^®^ cotton swab (Sarstedt, Nümbrecht, Germany) and a 3% citric acid solution as previously described [50]. Throughout the procedure, the owner sat next to the dog to minimize the cortisol response to adverse situations [72,73,74].

### 2.6. Settings

The study was performed in two dedicated rooms (waiting and examination room) where there were only the dog, the owner, and the two investigators, A, and B or C. Each evaluation took place in two main phases, in the two respective rooms. The floors in both rooms and the examination table were washed using a detergent with surfactants and after every examination by using an enzymatic cleanser (Septozym^®^, Nuova Farmec, Settimo di Pescantina, Italy) to eliminate pheromone pollution.

#### 2.6.1. Waiting Room

The owner spread the DAP or placebo gel (one puff) on his/her hands right after entering the waiting room, until completely absorbed. Then, he stroked the whole body of the dog at least three times and then allowed the animal to move around on a lose leash for 15 min. The last three minutes of this interval were videotaped for the behavioral analysis. The dog was then subjected to the collection of saliva, followed by blood pressure and heart rate measurement. The activities completed in the waiting room are summarized in Table 5.

#### 2.6.2. Examination Room

The investigator performing the physical examination spread the same gel used by the owner on her hands about 5 min before coming in contact with the dog. The owner entered the room with the dog on a leash and then the investigator collected a cursory medical history for about 2 min. After that, the owner placed the dog on the examination table and removed the leash. The physical examination was completed in about 10 min, without any physical restraint, according to the following standardized pattern: head inspection and palpation; forelegs inspection and palpation, including the prescapular and axillary lymph nodes; evaluation of the skin and subcutaneous panniculus, including the skin elasticity test; popliteal lymph nodes palpation; hindlimbs inspection and palpation, including popliteal lymph nodes; perineum inspection; vulva/foreskin inspection; arterial pulse measurement (30 s); respiratory rate measurement (1 min); heart auscultation (1 min); lung auscultation (about 5 breaths for each side of the chest); abdominal palpation; rectal temperature measurement (1 min). The temperature measurement was performed at the end of the examination to avoid influencing the data collected in the previous phases of the clinical examination (heart rate and stress-related behaviors scores) [75]. The first three minutes on the examination table were videotaped for the behavioral analysis. During the physical examination, the owner stayed near the examination table without talking. However, if the dog showed signs of anxiety, the owner could comfort him/her and help the investigator with gentle restraint, if necessary. Punishment was not allowed for the duration of the entire procedure [76]. At the end of the physical examination, the dog was lifted from the table by the owner and placed on the floor. A second blood pressure measurement was then taken. Finally, a second saliva sample was collected. The activities carried out in the examination room are summarized in Table 6.

#### 2.6.3. Laboratory Procedures

After saliva collection, the Salivette tubes were centrifuged (3500× *g*, 10 °C for 15 min) and the saliva samples were stored at −20 °C [20]. After thawing, saliva samples were assayed for salivary cortisol using a highly sensitive enzyme immunoassay kit (Salimetrics^®^ Salivary Cortisol ELISA Kit, Salimetrics, State College, PA, USA) [50].

#### 2.6.4. Statistical Analysis

Data analysis was realized thanks to SAS 9.4 software Copyright (c) 2002–2012 by SAS Institute Inc., Cary, NC, USA. The significance threshold was fixed at 5%. Data from the waiting room and those from the examination room were analyzed independently. Effects of treatment were analyzed using mixed models in which the dog was considered as a random effect. More specifically, for continuous data, general linear mixed models (GLMM) were performed after the verification of residues normality. For variables expressed as frequencies, models for count data were used. The Poisson mixed model was applied on data as first intention. The dispersion was then evaluated. When overdispersion was detected, the negative binomial model was preferred. Some behaviors were performed by a small number of dogs during the trial. This resulted in a higher number of zero data than would normally be expected in the conventional models for count data. In this case, mixed zero-inflated Poisson (ZIP) or mixed zero-inflated negative binomial (ZINB) models were adopted depending on the dispersion of the data. For the subjective rating scales, mixed multinomial models for ordinal data were performed. To validate the study design which was a 2 x 2 cross-over, the carry-over effect was tested by including the sequence of administration of the treatments as an effect to test in addition to the treatment effect. When the sequence was significant, a carry-over effect was detected. In that case, only data from the first period were analyzed, which led to the analysis on two parallel groups, and in that case, the dog was not considered as a random effect anymore. Missing data were removed from the analysis.

## 3. Results

### 3.1. Animals

Twenty-eight owned dogs were exposed once to DAP and once to placebo: 19 pure-breeds and nine cross-breeds, 19 females (13 intact and six spayed) and six males (eight intact and one neutered), aged between 14 and 108 months (mean: 54.71; SD: 28.75), whose weight ranged from 1.1 to 40 kg (median: 15; 25° percentile: 6.225; 75° percentile: 33.5). One dog was excluded for bite attempts during the examination.

### 3.2. Effects of Treatment in the Waiting Room

A carryover effect was detected for sitting (DF = 24; t = −2.88; *p* = 0.01; ZIP). For this behavior specifically, only data from period 1 were included in the analysis of the treatment effect. For all the other parameters, no carry-over effect was detected. All results are summarized in Table 7.

#### 3.2.1. Behavioral Parameters

Significant differences were detected between the treatments, DAP or placebo, concerning the patterns panting (*p* = 0.027) and lip licking (*p* = 0.018): the duration of panting was significantly higher in presence of DAP, while the frequency of lip licking was significantly lower in presence of DAP. The remaining behavioral patterns were not significantly affected by the treatments. No significant differences were found in behavioral categories. However, a trend towards significance was found for the behavioral category “low body postures without *sitting*” (*p* = 0.058), with lower values in presence of DAP.

#### 3.2.2. Physiological Autonomic Parameters

No significant differences were found in heart rate and systolic blood pressure between the treatments.

#### 3.2.3. Physiological Neuroendocrine Parameters

No significant differences were found in salivary cortisol concentrations between the treatments. The salivary cortisol values of dogs exposed to DAP ranged from 0.77 µg/dL to 4.77 µg/dL and in dogs exposed to placebo from 0.80 µg/dL to 4.59 µg/dL.

### 3.3. Effects of Treatment in the Examination Room

A carryover effect was detected for the respiratory rate (DF = 26.3; F = 5.1; *p* = 0.03, GLMM). For this parameter specifically, only data from period 1 were included in the analysis of the treatment effect. For all the other parameters, no carry-over effect was detected. All results are summarized in Table 8.

#### 3.3.1. Behavioral Parameters

No significant differences were found in behavioral categories and patterns between the treatments.

#### 3.3.2. Physiological Autonomic Parameters

No significant differences were found in heart rate, systolic blood pressure, respiratory rate, and rectal temperature between the treatments.

#### 3.3.3. Physiological Neuroendocrine Parameters

No significant differences were found in salivary cortisol concentrations between the treatments. The salivary cortisol values of dogs exposed to DAP ranged from 0.65 µg/dL to 4.97 µg/dL (Mean: 2.29; SD: 1.34) and in dogs exposed to placebo from 0.65 µg/dL to 4.69 µg/dL (Mean: 2.25; SD: 1.36).

#### 3.3.4. Subjective Rating Scales

No significant differences were found in stiffness, fear expressed by the patient, and difficulty in performing physical examination between the treatments.

## 4. Discussion

This study shows that exposure to this DAP formulation significantly influences a small set of oral behaviors associated with the stress response of dogs in the waiting room, but it does not affect the behavioral and physiological stress responses evoked by a routine physical examination in the examination room.

### 4.1. Effect of DAP on Behavioral Parameters

In the waiting room, lip or nose licking was significantly less frequent in the presence of DAP, while the duration of panting was significantly lower in the presence of the placebo. In addition, a result close to statistical significance was found for the behavioral category “low body postures without *sitting*”, with lower scores in the presence of DAP. Lip/nose licking is a typical appeasement gesture in dogs [66,77,78] that is used to attempt to de-escalate a threat or to express a conflictive situation [78,79], and it is considered a behavioral sign of acute stress [7,77]. Appeasing behaviors in the category “Appeasement Gesture”, which include also lip/nose licking, were most frequently shown in the waiting room, mirroring the elevated frequency with which these behaviors are seen in dogs. Low body postures are attributable to fear, attempts at pacification, or even to a stressful interaction [66,78]. Furthermore, it has been shown that a moderate lowering of the posture together with high levels of oral behaviors may be shown in the case of moderate stress elicited by a social setting [7]. Both lip/nose licking and low body postures are signals of visual communication, which is particularly useful in situations of close social proximity, since it allows a quick passage of the message from the emitter to the receptor, thus eliciting a fast response [78].

The fact that, in this study, exposure to DAP decreased the occurrence of lip/nose licking (*p* < 0.050) and low body posture (*p* = 0.058) in the same standardized clinical setting, the waiting room, suggests that the treatment might have contributed to the reduction of the stress resulting from staying in the waiting room. However, the increase in the duration of panting in dogs exposed to the DAP seems to be in disagreement with the previous results. This apparent contradiction may be explained with the following considerations. Among the stress-related factors significantly influenced by the treatment, panting is more likely to be influenced by environmental factors that we did not control, like temperature. The dogs may have received the DAP treatment on days with a higher temperature than the days when they received the sham treatment. Moreover, the neurophysiology of panting within the stress response is not well understood, as it can be a thermoregulatory mechanism, in the case of thermal stress [4,80,81], or be part of a wider autonomic response to stress. Not much is known about how the sympathetic and parasympathetic branches of the ANS modulate panting when the stress response is activated. We can consider the example of the cardiac frequency, another stress marker associated with the cardiorespiratory system. We know that an increase of its frequency is a less reliable marker than the heart rate variability, which assesses the action of both the sympathetic and the parasympathetic system. Something similar may be true for panting, which could vary in unexpected ways depending on the context and stimuli involved in the stress response. We may therefore hypothesize that a decrease of panting in the absence of exposure to DAP is due to an increase of the vagal tone involved in a freeze stress response [82,83], and the application of the appeasing pheromone decreases the vagal tone and favors thermoregulation via panting. Lastly, we should keep in mind the assumed 5% chance that the results observed in this study for all variables showing significant changes may be observed if the null hypothesis is true (type I error).

The absence of any measurable effect of the DAP in the examination room, including the subjective behavioral rating scales, can derive from a major stress level during the physical examination that has been documented in the published scientific literature [27,29,30,31,32,84]. Moreover, the restraining and handling during the physical examination may limit the opportunities to display common coping behavior signs [85].

The results of the present study are In agreement with a clinical study aimed at evaluating the effect of DAP on the behavior and emotional state of dogs undergoing a clinical examination that were enrolled based on fear- and anxiety-related behaviors exhibited during previous veterinary visits [49]. In that study, it was found that dogs that received DAP were less anxious and more relaxed in the waiting room, while no significant difference was found while the dogs were examined on the table.

### 4.2. Effect of DAP on Autonomic Parameters

The comparison between the two treatments did not show any statistically significant effect of the DAP on the heart rate and systolic blood pressure, neither in the waiting room nor in the examination room. These results can be explained by two not mutually exclusive hypotheses: the effect, if any, of the DAP on the sympathetic response, assessed using biomarkers characterized by high individual variability [2,3,86,87], was not strong enough to be appreciated in this study; or the physiological stress experienced by the dogs was too high to be antagonized by DAP. In this regard, the procedures used to measure these biomarkers may represent a remarkable source of stress themselves [2]. This is true in particular for the measurement of blood pressure.

To the authors’ knowledge, there are no published studies assessing the action of DAP on the autonomic stress response in dogs undergoing veterinary examination. However, the results of the present study are in agreement with those that emerged from a similar study that tested the efficacy of a DAP diffuser on heart rate and eye and ear temperature of healthy dogs separated from the owner in a laboratory setting, concluding that the treatment did not markedly affect any of these parameters in the absence of the owner [88]. Although different experimental settings and methods of measurement have been used, both studies agree that the autonomic response in dogs facing a stressful situation may not benefit from treatment with DAP.

### 4.3. Effect of DAP on Neuroendocrine Parameters

The comparison between the two treatments did not show any statistically significant difference in salivary cortisol, neither in the waiting room nor in the examination room, with identical mean values regardless of the treatment. These results could be explained by a lack of sensitivity of cortisol to treatment with DAP or to its high individual variability due to genetic, experiential, and environmental factors [2,21,89,90]. The cortisol values of the present study ranged from 0.77 µg/dL to 4.77 µg/dL, in line with the range of salivary cortisol provided by a published meta-analysis, confirming the variability of this parameter in the canine species [89].

To the authors’ knowledge, there are no published studies assessing the action of DAP on the neuroendocrine stress response in dogs undergoing veterinary examination. However, our results agree with a study that assessed the cortisol response to DAP in dogs undergoing elective surgery [50], using the same collection and laboratory procedures, but different animal management. In this case, the salivary cortisol was also unaffected by treatment. Based on the results of both studies, cortisol does not seem to be adequate for measuring the acute stress response elicited by a veterinary setting.

### 4.4. Study Limitation

Given the high individual variability of the tested parameters, the small sample size of this study may have made the effects of DAP undetectable. The use of citric acid as a salivary stimulant is currently controversial because it could lead to changes in saliva composition, affecting the assay results [21,89,91].

## 5. Conclusions

The results of this study show a significant but small effect of the dog appeasing pheromone on behavioral markers typically associated with the stress response. This effect was limited to the time the dogs spent in the waiting room and focused on the oral behaviors *lip licking* and *panting*. While the frequency of lip licking decreased as expected in dogs exposed to DAP, the duration of panting increased in these animals. This apparent incongruence may be due to the influence of environmental factors, to the poorly understood autonomic control of thermoregulation and panting, or to a type I error that should be considered for all variables showing a significant change.

Considering with the body of evidence already available about the use of appeasing pheromones in clinical settings, our findings suggest that the use of dog appeasing pheromones might contribute to the control of the stress experienced in the veterinary waiting room. Further studies are necessary to confirm how the time of administration of DAP gel influences the stress response. Administering the gel before a dog reaches the veterinary facility might improve the response to the appeasing pheromones.

The behavioral markers were the most sensitive to estimate and treat the stress response elicited by the veterinary visit, so veterinarians should be encouraged to focus on the dog’s body language since it provides useful and real-time information on how the animal is adapting at any given moment, thus allowing early and more effective interventions.

## Figures and Tables

**Table 1:** Composition of the two treatments.

Composition	Test Product (%)	Placebo (%)
DAP 100%	1	0
Ethanol	69	70
Gelling agent	1.5	1.5
Glycerine	3.0	3.0
Water	25.5	25.5

**Table 2:** Ethogram used for behavioral analysis. Since some behaviors occurred only in one of the two rooms set up for observation, the ethogram was integrated by specifying, with an X, which was the reference setting for each behavioral category.

Behavioral Categories	Definitions of Individual Patterns	Waiting Room	Examination Room
*Explorative behavior*			
Olfactory exploration	moving the nose along objects/people or exhibiting clear sniffing movements	X	X
Visual exploration	visual scanning or observation of the environment	X	X
*Appeasement gesture*			
Lip or nose licking	running the tongue over the lips or the nose	X	X
Paw lifting	raising a forepaw into a position of approximately 45°	X	X
Turning head	the dog rotates its head by more than 45		X
Blinking	opening and closing eyes repeatedly	X	X
*Social approach behavior*			
Oriented to investigators	sitting, standing or lying down staring fixedly at the investigators, regardless of whether the behavior was reciprocated	X	
Oriented to owner	sitting, standing or lying down staring fixedly at the owner, regardless of whether the behavior was reciprocated	X	X
Tail wagging	repetitive motion of the tail	X	X
Low body postures *			
Lowered ears	the ears are positioned backward	X	X
Lowered tail/Tail between legs	a lowered position of the tail, compared to the breed-specific position shown by the dog under neutral conditions/the tail is curled forward between the hind legs	X	X
Lowered head	a lowered position of the head, compared to the position shown by the dog under neutral conditions		X
Sitting	sitting on the ground with the pads of the front paws in contact with the floor and forelimbs straight	X	X
*Avoidance behavior*			
Oriented to door	sitting, standing or lying down staring fixedly at the door, either when close to it or from a distance	X	
Trying to jump from the table			X
*Further stress indicating activities*			
Panting	increased frequency of inhalation and exhalation in combination with the opening of the mouth	X	X

* Since a very low occurrence of the pattern *sitting* was recorded in the behavioral category “low body postures”, it was decided to create a sub-category of this category that did not include it (“low body postures without *sitting*”) in regards to the waiting room.

**Table 3:** Behavioral patterns evaluated for frequency (^F^) or duration (^D^).

Waiting Room	Examination Room
Olfactory exploration ^D^	Visual exploration ^D^
Visual exploration ^D^	Sitting ^D^
Sitting ^D^	Lip or nose licking ^F^
Lip or nose licking ^F^	Lowered ears ^D^
Lowered tail/tail between legs ^D^	Lowered tail/tail between legs ^D^
Oriented to door ^D^	Lowered head ^D^
Panting ^D^	Panting ^D^

**Table 4:** Likert-type rating scales about veterinarian’s perception during the physical examination.

Scale	Scoring
Stiffness of the patient	0 1 2 3
Fear expressed by the patient (e.g., trembling)	0 1 2 3
Difficulty in performing the physical examination	0 1 2 3

**Table 5:** Data collection program in the waiting room.

Sequence of Events	Activities	Evaluated Parameters
T_1_	Video-recording of the behavior	Behavioral categories and patterns
T_2_	Saliva collection	Salivary cortisol
T_3_	Blood pressure and arterial pulse detection	Systolic blood pressure, heart rate

**Table 6:** Data collection program in the examination room. *: first three minutes of the physical examination.

Sequence of Events	Activities	Evaluated Parameters
T_1_	Video-recording of the behavior *	Behavioral categories and patterns
	Physical examination	Heart rate, respiratory rate, rectal temperature, stiffness, fear expressed by the patient, difficulty in the execution of the examination
T_2_	Blood pressure detection	Systolic blood pressure
T_3_	Saliva collection	Salivary cortisol

**Table 7:** Statistical evaluation of data collected in the waiting room from 28 dogs when exposed to DAP or placebo: descriptive statistic (mean and standard deviation) and comparison of the effects of DAP versus placebo.

Behavioral Categories and Patterns	DAP	Placebo	Model	Treatment Effect
*Explorative behavior*	2.0 (1.1)	1.7 (0.9)	ZIP	DF = 24; t = 1.09; *p* = 0.29
Olfactory exploration	10.9 (10.3)	11.6 (12.6)	ZINB	DF = 23; t = 0.16; *p* = 0.87
Visual exploration	6.7 (6.1)	5.9 (4.0)	ZINB	DF = 24; t = 0.50; *p* = 0.62
*Appeasement gesture*	1.1 (0.8)	1.2 (0.9)	Poisson	DF = 22; F = 0.05; *p* = 0.82
Lip or nose licking	1.9 (1.1)	4.2 (3.7)	ZIP	DF = 24; t = −2.52; *p* = 0.02
*Social approach behavior*	1.4 (1.1)	1.5 (1.1)	Poisson	DF = 23; F = 0.03; *p* = 0.86
*Low body posture* *s*	1.3 (1.1)	1.4 (0.8)	ZIP	DF = 24; t = 0.0; *p* = 1.00
*Low body posture* *s* *without sitting*	0.9 (0.5)	2.0 (1.0)	ZIP	DF = 24; t = −1.99; *p* = 0.06
Lowered tail/tail between legs	17.9 (18.1)	20.3 (17.0)	ZINB	DF = 24; t = −0.30; *p* = 0.77
Sitting	3.3 (9.4)	6.3 (10.8)	ZINB	DF = 24; t = 0.58; *p* = 0.45
*Avoidance behavior*	2.6 (1.8)	2.6 (1.5)	ZIP	DF = 24; t = 0.05; *p* = 0.96
Oriented to door	12.5 (14.3)	10.8 (10.2)	ZINB	DF = 24; t = −0.20; *p* = 0.84
*Further stress indicating activities*	4.7 (1.4)	4.5 (1.6)	ZIP	DF = 24; t = −0.16; *p* = 0.87
Panting	28.3 (13.6)	23.8 (10.7)	ZIP	DF = 24; t = 2.35; *p* = 0.03
*Physiological autonomic parameters*				
Heart rate	99.4 (18.8)	93.8 (22.4)	GLMM	DF = 26; F = 2.28; *p* = 0.14
Systolic blood pressure (mm Hg)	135.1 (19.0)	138.6 (13.3)	GLMM	DF = 21.8; F = 1.13; *p* = 0.30
*Physiological neuroendocrine parameters*				
Salivary cortisol (µg/dL)	2.1 (1.2)	2.1 (1.0)	GLMM	DF = 21.2; F = 0.12; *p* = 0.73

Legend: ZIP = mixed zero-inflated Poisson; ZINB = mixed zero-inflated negative binomial; GLMM = general linear mixed models; DF = Degrees of freedom, F = Fisher’s statistic, t = Student’s statistic.

**Table 8:** Statistical evaluation of data collected in the examination room from 28 dogs when exposed to DAP or placebo: descriptive statistic (mean and standard deviation) and comparison of the effects of DAP versus placebo.

Behavioral Categories and Patterns	DAP	Placebo	Model	Treatment Effect
*Explorative behavior*	2.2 (1.0)	2.0 (1.2)	Poisson	DF = 25; F = 0.22; *p* = 0.64
Visual exploration	15.0 (9.2)	12.2 (9.4)	Neg. Binomial	DF = 25; F = 1.1; *p* = 0.31
*Appeasement gesture*	1.9 (0.6)	2.1 (0.5)	Poisson	DF = 24; F = 0.49; *p* = 0.49
Lip or nose licking	4.1 (5.6)	5.2 (7.1)	Poisson	DF = 25; F = 1.1; *p* = 0.31
*Social approach behavior*	1.0 (0.8)	1.1 (0.8)	Poisson	DF = 24; F = 0.13; *p* = 0.72
*Low body postures*	2.4 (1.1)	2.3 (0.9)	Poisson	DF = 25; F = 0.04; *p* = 0.85
Lowered ears	42.7 (18.9)	40.8 (23.7)	ZIP	DF = 26; t = −0.54; *p* = 0.59
Lowered tail/tail between legs	44.9 (21.0)	48.1 (18.8)	ZINB	DF = 26; t = −0.22, *p* = 0.83
Lowered head	13.2 (14.2)	9.9 (10.0)	ZINB	DF = 26; t = 0.65; *p* = 0.52
Sitting	18.5 (11.5)	16.9 (11.0)	ZIP	DF = 26; t = −1.09; *p* = 0.29
*Avoidance behavior*	1.3 (0.6)	1.6 (0.8)	ZIP	DF = 26; t = −0.00; *p* = 1.00
*Further stress indicating activities*	1.3 (0.9)	1.1 (1.8)	-	-
Panting	3.0 (8.2)	6.7 (11.6)	-	-
*Physiological autonomic parameters*				
Heart rate Systolic blood pressure Rectal temperature Respiratory rate	101.1 (22.2) 132.7 (16.7) 38.4 (0.5) 39.0 (21.8)	98.1 (24.8) 134.2 (19.4) 38.5 (0.5) 43.9 (26.8)	GLMM GLMM GLMM Wilcoxon	DF = 26; F = 0.74; *p* = 0.40 DF = 24.6; F = 0.11; *p* = 0.74 DF = 23.6; F = 3.27; *p* = 0.08 Z = −1.48; *p* = 0.14
*Physiological neuroendocrine parameters*				
Salivary cortisol (µg/dL)	2.3 (1.3)	2.3 (1.4)	GLMM	DF = 19.7; F = 0.09; *p* = 0.77
*Subjective scales*				
Stiffness				
0	16 (57.1)	15 (55.6)	Multinomial	DF = 23; F = 0.41; *p* = 0.53
1	4 (14.3)	9 (33.3)		
2	7 (25.0)	2 (7.4)	l	
3	1 (3.6)	1 (3.7)		
Fear expressed by the patient (e.g., trembling)				
0	17 (60.7)	18 (66.7)	Multinomial	DF = 23; F = 0.41; *p* = 0.53
1	5 (17.9)	1 (3.7)		
2	4 (14.3)	5 (18.5)		
3	2 (7.1)	3 (11.1)		
Difficulty in the execution of the examination				
0	19 (67.9)	17 (63.0)	Multinomial	DF = 23; F = 1.45; *p* = 0.24
1	3 (10.7)	6 (22.2)		
2	6 (21.4)	2 (7.4)		
3	0 (0.0)	2 (7.4)		

Legend: ZIP = mixed zero-inflated Poisson; ZINB = mixed zero-inflated negative binomial; GLMM = general linear mixed models; DF = Degrees of freedom, F = Fisher’s statistic, t = Student’s statistic.

## Data Availability

The data presented in this study are available on request from the corresponding authors.
